# Length of paternal lifespan is manifested in the DNA methylome of their nonagenarian progeny

**DOI:** 10.18632/oncotarget.5905

**Published:** 2015-09-30

**Authors:** Saara Marttila, Laura Kananen, Juulia Jylhävä, Tapio Nevalainen, Antti Hervonen, Marja Jylhä, Mikko Hurme

**Affiliations:** ^1^ Department of Microbiology and Immunology, School of Medicine, University of Tampere, Tampere, Finland; ^2^ Gerontology Research Center, Tampere, Finland; ^3^ School of Health Sciences, University of Tampere, Tampere, Finland; ^4^ Institute for Advanced Social Research, University of Tampere, Tampere, Finland; ^5^ Fimlab Laboratories, Tampere, Finland

**Keywords:** lifespan, longevity, DNA methylation, methylome, intergenerational inheritance, Gerotarget

## Abstract

The heritability of lifespan is 20-30%, but only a few genes associated with longevity have been identified. To explain this discrepancy, the inheritance of epigenetic features, such as DNA methylation, have been proposed to contribute to the heritability of lifespan.

We investigated whether parental lifespan is associated with DNA methylation profile in nonagenarians. A regression model, adjusted for differences in blood cell proportions, identified 659 CpG sites where the level of methylation was associated with paternal lifespan. However, no association was observed between maternal lifespan and DNA methylation. The 659 CpG sites associated with paternal lifespan were enriched outside of CpG islands and were located in genes associated with development and morphogenesis, as well as cell signaling. The largest difference in the level of methylation between the progeny of the shortest-lived and longest-lived fathers was identified for CpG sites mapping to *CXXC5*. In addition, the level of methylation in three Notch-genes (*NOTCH1*, *NOTCH3* and *NOTCH4*) was also associated with paternal lifespan.

There are implications for the inheritance of acquired traits via epigenetic mechanisms in mammals. Here we describe DNA methylation features that are associated with paternal lifespan, and we speculate that the identified CpG sites may represent intergenerational epigenetic inheritance.

## INTRODUCTION

The heritability of lifespan (age at death) has been estimated to be approximately 20-30%, and it has been shown to increase with advancing age. Healthy aging is also heritable, and the offspring of long-lived parents show delayed onset of aging-associated diseases [1, 2, 3, 4]. Much of the research studying the heritability of lifespan has focused on extreme age (nonagenarians, centenarians, supercentenarians), but recently it has been shown that every decade of parental age after the age of 65 reduces the mortality and incidence of cancer of their offspring [5].

Even though the heritability of the lifespan is acknowledged, only one genomic locus (on chromosome 3) and a few genetic variants, such as in *APOE* and *FOXO3,* have consistently been shown to be associated with longevity. Data regarding other genomic loci and genes, including *CETP*, *HSF2* and *MTP*, have been inconsistent between studies [3]. Therefore, in addition to disease susceptibility alleles, rare genetic variants and environment-genome interactions, epigenetic mechanisms such as DNA methylation may be mediating the heritability of lifespan.

Changes in DNA methylation are associated with aging and many aging-associated diseases, such as cancer, Alzheimer's disease and type 2 diabetes [6]. These changes include global hypomethylation and site-specific hypermethylation [7, 8, 9], and both tightly regulated and environmental or stochastic effects have been reported [10, 11, 12]. Aging-associated hypomethylation has been shown to be delayed in the offspring of centenarians [13].

The role of transgenerational epigenetic inheritance in the heritability of acquired traits has been discussed in the literature. In mice and rats, there is evidence that environmental exposure (for example, to vinclozolin and ethanol) causes phenotypic effects and changes in somatic and sperm DNA methylation, and these effects have been shown to be transmitted to the F4 generation [14, 15, 16, 17]. In a majority of the studies, the inheritance of epigenetic features is affected by the sex of the parent or progeny [15, 16, 18].

In humans, the environmental conditions experienced during early childhood or fetal development have been shown to link to epigenetic features, typically DNA methylation, in adulthood. For example, the progeny of mothers who experienced famine during early pregnancy are more prone to obesity and raised blood lipids, and the methylation status of the *IGF2* gene is affected in these progeny [19, 20]. In addition, childhood abuse has been associated with alterations to DNA methylation in middle-aged men [21]. In some cases, the environmental factors (e.g. nutrition, tobacco smoking, and betel quid chewing) experienced by fathers or grandfathers have been shown to affect the phenotype of their sons or grandsons (e.g. increased risk of diabetic death and increased adiposity). It is suspected that these traits are inherited via epigenetic mechanisms [18].

The effect of length of parental lifespan on the DNA methylation profile of progeny has not been previously studied. Here, we sought to identify DNA methylation patterns that are associated with maternal or paternal lifespan (age at death) to determine whether this trait manifests in the DNA methylome of progeny. DNA methylation profiles that are common among the progeny of longer-living parents may be components that are partially responsible for the heritability of lifespan.

## RESULTS

### Long-living fathers, long-living siblings

The study population consisted of 90 nonagenarians who participated in the Vitality 90+ study cohort of 2010 [8, 22]. In the regression model used to identify CpG sites associated with parental age, parental age was used as a continuous variable. However, for group comparisons, the population was divided into three groups according to paternal (FI (shortest-living fathers), FII, FIII (longest-living fathers)) and maternal age (MI (shortest-living mothers), MII, MIII (longest-living mothers)). See Table 1 for distribution of parental ages.

**Table 1:** Grouping of study population according to paternal and maternal lifespan

	n	Age of father at death	Age of mother at death
Whole population	90	40-103 (67)	40-101 (79.5)
Group FI	32	40-60 (55)	
Group FII	30	61-75 (67.5)	
Group FIII	28	77-103 (83)	
Group MI	32		40-72 (58)
Group MII	32		75-83 (80)
Group MIII	26		84-101 (88.5)

For group comparisons, the population was divided according to paternal and maternal lifespan. Presented here are the age range and (median) age at death for fathers and mothers.

We found that group FIII (progeny of the longest-living fathers) had more long-living siblings (siblings living over 85 years) compared to group FI (Mann-Whitney U-test *p* = 0.004). This difference remains statistically significant when considering siblings over 75 years (*p* = 0.006) or siblings over 80 years (*p* = 0.006). The lifespan of the mother had no effect on the number of long-living siblings (comparison between groups MIII and MI: for siblings over 85 years of age, *p* = 0.148, for siblings over 80 years, *p* = 0.338 and for siblings over 75 years, *p* = 0.242).

Paternal lifespan was not correlated with maternal lifespan (Spearman's rho = 0.159, *p* = 0.135) or with paternal age at conception (data on paternal age at conception available only for a subset of the population (*n* = 21), Spearman's rho = −0.252, *p* = 0.271). In addition, paternal lifespan was not associated with the socio-economic status of offspring.

### Association of paternal age with DNA methylation profile

The DNA methylation profile was determined with Illumina Infinium HumanMethylation450 BeadChip from peripheral blood mononuclear cells. We identified 659 CpG sites where the level of methylation was associated with paternal lifespan (regression model *p*-value < 0.05 (BH-corrected), Δβ between group FIII and FI >1%, see Supplementary Table 1). Of the CpG sites associated with paternal lifespan, higher paternal age was associated with decreasing level of methylation in 423 (64%). There were no CpG sites where the level of methylation was associated with maternal lifespan. It is noteworthy that both the number of long-living siblings and the DNA methylation profile were associated with paternal lifespan, but not with maternal lifespan.

**Figure 1:**
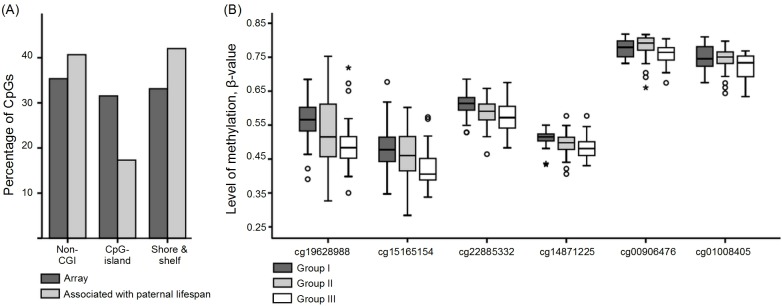
Location of CpG sites associated with paternal lifespan and methylation level of CXXC5 **A.** Location of CpG sites associated with paternal lifespan with regard to CpG islands. There were fewer than expected CpG sites found in CpG islands (hypergeometric test *p* < 0.05). **B.** Differences in the level of methylation in *CXXC5*. Level of methylation in each CpG site is presented for each group (Group I, progeny of shortest-lived fathers, Group III, progeny of longest-lived fathers).

The CpG sites that were associated with paternal age were not enriched in any particular chromosome or gene location (hypergeometric test p>0.05). However, there were fewer than expected CpG sites in CpG islands (hypergeometric test *p* < 0.05, see Figure 1A).

Because a small number of DNA methylation changes in key genes that are involved in a given biological process can regulate the whole process or pathway (without DNA methylation changes to other genes), we wanted to investigate the identified top hits more closely. We defined the top hits as CpG sites with >5% difference in Δβ between groups FIII and FI or CpG sites that were located in a gene that harbored at least two CpG sites associated with paternal age. There were 65 CpG sites located in 46 different genes with Δβ>5%, and 31 additional genes had at least two CpG sites that were associated with paternal lifespan. Combined, there were 146 CpG sites and 77 genes that were further characterized (Supplementary Table 2). Among all CpG sites associated with paternal age, there were more sites where the level of methylation decreased as paternal age increased. This trend was even more pronounced among the top hits, where 116 out of 146 sites (79%) showed decreasing methylation levels with increasing paternal age.

*CXXC5* (CXXC finger protein 5) was the most affected gene, harboring 6 CpG sites where the level of methylation was associated with paternal lifespan, and in all of these CpG sites, higher paternal age was associated with a decreased level of DNA methylation (Figure 1B). All genes with 3 or more CpG sites associated with paternal age are presented in Table 2. The largest Δβ between group FIII and group FI were observed at cg19628988 (*CXXC5*, Δβ = −0.082), cg12076931 (*NOTCH1*, Δβ = −0.080), cg23644389 (Δβ = 0.072) and cg24607398 (*EPM2AIP1*, Δβ = 0.069) (Table 3). In addition to *NOTCH1*, *NOTCH4* also included a CpG site with a Δβ>5% (cg06023661, Δβ = −0,066) and both genes harbored an additional CpG site (in *NOTCH1*, cg13861904, Δβ = −0.042 and in *NOTCH4*, cg06815976, Δβ = −0.042). *NOTCH3* also harbored two CpG sites where the level of methylation was associated with paternal lifespan (cg27320207, Δβ = −0.038 and cg26880200, Δβ = 0.020).

**Table 2:** Genes with the largest number of CpG sites associated with paternal lifespan

	n(CpG)	ID	*p* -value (BH-corrected)	Δβ
*CXXC5*	6	cg19628988	0.049	−0.082
		cg15165154	0.023	−0.072
		cg22885332	0.049	−0.042
		cg14871225	0.040	−0.034
		cg00906476	0.046	−0.015
		cg01008405	0.032	−0.012
*COL11A2*	4	cg13683990	0.042	−0.025
		cg21232625	0.042	−0.024
		cg25459558	0.028	−0.023
		cg02266086	0.046	−0.020
*KCNS1*	4	cg25353142	0.023	−0.033
		cg27634724	0.025	−0.023
		cg07589968	0.038	−0.021
		cg06193004	0.021	−0.017
*BID*	3	cg03433260	0.042	−0.017
		cg20234121	0.044	−0.014
		cg01280609	0.025	−0.013
*FGR*	3	cg09845000	0.029	−0.046
		cg09370867	0.030	−0.046
		cg13448978	0.046	−0.042
*LOC283050*	3	cg24658487	0.021	−0.041
		cg22890825	0.046	−0.035
		cg06891775	0.049	−0.018

In total, 42 genes harbored more than one affected CpG site (see Supplementary Table 2) and 6 genes contained three or more CpG sites associated with paternal lifespan. Δβ refers to the difference in methylation level between group FIII and group FI.

**Table 3:** CpG sites associated with paternal lifespan that had the largest Δβ between group FIII and group FI

Gene	ID	*p*-value (BH-corrected)	Δβ
*CXXC5*	cg19628988	0.048	−0.082
*NOTCH1*	cg12076931	0.032	−0.080
*KRT27*	cg10747531	0.032	−0.077
na	cg11284147	0.047	−0.077
*CXXC5*	cg15165154	0.023	−0.072
*MPZL1*	cg04846203	0.035	−0.067
*NOTCH4*	cg06023661	0.038	−0.066
*UEVLD*	cg15846482	0.033	−0.065
*SORT1*	cg02175308	0.028	−0.065
*DAP*	cg14129473	0.032	−0.064
*MORC2*	cg23825480	0.047	0.055
*RRAD*	cg06410849	0.032	0.056
*RESP18*	cg19020434	0.032	0.057
*ITPKB*	cg23717186	0.037	0.059
na	cg00248242	0.041	0.059
*CPA5*	cg22664614	0.039	0.059
na	cg14828411	0.040	0.060
*GULP1*	cg16947583	0.034	0.062
*EPM2AIP1*	cg24607398	0.023	0.069
na	cg23644389	0.045	0.072

For all CpG sites associated with paternal lifespan, see Supplementary Table 1. na = no gene annotation available.

### Pathways

The 659 CpG sites associated with paternal lifespan were located in 422 different genes. Cellular processes and signaling pathways associated with the identified genes were searched using QIAGEN's Ingenuity^®^ pathway analysis (IPA) [23] and GOrilla [24,25]. We identified only one canonical pathway, B cell receptor signaling, that was associated with the identified genes when p-values were corrected for multiple testing (BH-corrected *p*-value < 0.05). Using GO term analysis, we identified 35 enriched GO process terms (BH-corrected *p*-value < 0.05) that were associated with genes harboring the CpG sites that were associated with paternal lifespan. The identified GO process terms were associated with development and morphogenesis and with cell signaling (Table 4).

**Table 4:** GO process terms associated with genes where methylation level is associated with paternal lifespan

GO Term	Description	*p*-value (BH-corrected)
GO:0048523	negative regulation of cellular process	0.011
GO:0010646	regulation of cell communication	0.012
GO:0022603	regulation of anatomical structure morphogenesis	0.013
GO:0023051	regulation of signaling	0.014
GO:0040012	regulation of locomotion	0.016
GO:0044767	single-organism developmental process	0.016
GO:0009966	regulation of signal transduction	0.017
GO:0032502	developmental process	0.019
GO:0048519	negative regulation of biological process	0.022
GO:0030154	cell differentiation	0.022
GO:0009653	anatomical structure morphogenesis	0.024
GO:0051270	regulation of cellular component movement	0.024
GO:0050878	regulation of body fluid levels	0.024
GO:0050794	regulation of cellular process	0.025
GO:2000147	positive regulation of cell motility	0.025
GO:0044707	single-multicellular organism process	0.025
GO:0031325	positive regulation of cellular metabolic process	0.025
GO:0040017	positive regulation of locomotion	0.026
GO:0051239	regulation of multicellular organismal process	0.026
GO:0007165	signal transduction	0.026
GO:0090527	actin filament reorganization	0.026
GO:0048583	regulation of response to stimulus	0.026
GO:0009893	positive regulation of metabolic process	0.026
GO:0048522	positive regulation of cellular process	0.027
GO:0048856	anatomical structure development	0.027
GO:0030335	positive regulation of cell migration	0.027
GO:0051272	positive regulation of cellular component movement	0.028
GO:0048869	cellular developmental process	0.028
GO:0048518	positive regulation of biological process	0.029
GO:0032501	multicellular organismal process	0.030
GO:0007596	blood coagulation	0.032
GO:0050817	coagulation	0.033
GO:0007599	hemostasis	0.034
GO:0050789	regulation of biological process	0.035
GO:0065007	biological regulation	0.047

The 659 CpG sites associated with paternal lifespan were located in 422 different genes, and these genes were enriched to 35 GO process terms (Benjamini-Hochberg multiple testing corrected *p*-value of < 0.05).

In the GO term analysis for the top hits, no term reached multiple testing-corrected statistical significance (BH-corrected *p*-value < 0.05), but there was a trend toward developmental and signaling processes. Similarly, no significant canonical pathways were identified when multiple testing correction was used (BH-corrected *p*-value < 0.05). However, Notch-signaling was closest to the significance threshold (BH-corrected *p*-value = 0.084).

## DISCUSSION

Here, we report the identification of 659 CpG sites where the level of methylation was associated with length of paternal lifespan. These results were adjusted for differences in blood cell type percentages. We speculate that these sites may represent intergenerational epigenetic inheritance and that these methylation sites could be associated with heritability of lifespan.

### Cell signaling

*CXXC5*, a member of the small zinc finger protein family, contained 6 CpG sites where the level of methylation decreased as paternal lifespan increased. *CXXC5* negatively regulates Wnt/β-catenin signaling [26, 27, 28] and has been shown to be a mediator in BMP-signaling [29]. *CXXC5* has a role in normal and tumoral myelopoiesis [30] and in endothelial cell differentiation and migration and vessel formation [29]. The CXXC motif recognizes unmethylated CpG sites, and these proteins are involved in epigenetic modifications [31].

Six CpG sites in three Notch genes (*NOTCH1, 3* and *4*) were associated with paternal lifespan in our study. In addition, pathway analyses implied that Notch-signaling is associated with DNA methylation changes that are associated with paternal lifespan. The Notch-signaling pathway functions in various cell types and at various time points during development. Notch-signaling plays a role in development and organogenesis, and also in adult tissue maintenance and repair [32]. Notch-signaling has been associated with aging associated loss of muscle mass and function (sarcopenia). Impairments in Notch-signaling may be responsible for loss of myogenic potential in aged muscle. This association may also be due to an imbalance in Notch- and Wnt-signaling [32, 33, 34]. In addition, disruptions in Notch-signaling have been implicated in certain cancers and associated with Alzheimer's disease [32].

GO term analysis showed that signaling was affected, and B cell receptor signaling was also specifically identified as being associated with the identified genes in our study. In parallel with other changes in the immune system, the B cell pool goes through various changes during aging, and some of these changes have also been associated with adverse health outcomes [35, 36]. Our results imply that these changes can be partially regulated by DNA methylation. However, because we were unable to adjust the analysis for the proportion of B cells, this result may be due to differences in B cell proportions across study samples.

A previous study showed that genes that are hypomethylated in the offspring of nonagenarians (compared to progeny of non-long-lived parents) were also associated with signal transmission [13], and our own results show that GO process terms, such as regulation of cell communication (GO:0010646) and regulation of signaling (GO:0023051), are associated with genes that contain methylation sites where the level of methylation is associated with paternal lifespan (Table 4). A review by Carlson et al. [32] discussed the association between aging and changes in signaling intensities in various signaling pathways (for example Notch-, TGFβ- and Wnt-signaling). These pathways function in an intertwined network, and proper regulation is needed to balance signaling during development and adult tissue maintenance and repair.

### Location of CpGs associated with paternal age

Of the identified CpG sites associated with paternal lifespan, fewer were located in CpG-dense CpG islands than expected. They were instead enriched outside of CpG islands and in shores and shelves. It has been shown, in mice, that methylation level is associated with paternal environmental effects at CpG sites that are located in low-CG areas of the genome [37]. DNA methylation at CpG islands, and particularly at transcription start sites, is usually considered to be the more important regulator of gene expression [38], but the CpG-poor regions of the genome have also recently been proposed to be important for regulation [39, 40].

### Aging and longevity are linked with development

We found that methylation sites that were associated with paternal lifespan were enriched in genes associated with development and morphogenesis. Aging associated hypermethylation has also been shown to be enriched in genes associated with development and morphogenesis [8, 13, 41, 42, 43].

The roles of developmental or metabolic rates in aging and longevity have been extensively studied, and caloric restriction, body size, changes in insulin signaling and the mTOR-pathway also have implied associations with longevity [44, 45, 46]. Alterations in the epigenetic mechanisms that control developmental processes may also contribute to lifespan. The role of developmental programs in aging is also a component of the quasi-programmed hyperfunction theory of aging, which states that aging is the aimless continuation of a developmental program when it is no longer needed [47]. Because these developmental programs are needed early in life, large alterations to these pathways would likely be deleterious. Thus, we expect that the DNA methylation changes identified in this study have only small effects on lifespan.

### Mechanism of epigenetic inheritance

Both human and mouse studies have implied that certain traits acquired by a parent can be inherited by progeny, at least for one or two generations, and that some of these cases involve DNA methylation (see Introduction). The molecular mechanism explaining how inheritance through DNA methylation patterns occurs is still lacking, because the DNA methylome goes through two major reprogramming steps, first in the embryo and then in primordial germ cells [15, 48]. However, imprinted genes do have parent-of-origin-dependent expression patterns [49], and it has recently been shown that in mice, certain genomic regions at least partially avoid the reprogramming of the DNA methylome [50, 51]. Thus, transgenerational epigenetic inheritance is at least plausible in humans.

The DNA methylation features associated with paternal lifespan that were identified in this study may be intergenerationally inherited. However, we cannot exclude that the hereditary component may be another epigenetic feature, rather than DNA methylation, or traditional genetic element that contributes to the perceived DNA methylation pattern.

Both transgenerational epigenetic inheritance and the heritability of longevity and lifespan appear to be dependent on the sex of the parent and/or progeny, although reported results are inconsistent in the case of longevity and lifespan [5]. Our results show that the DNA methylation landscape and the number of longer-living siblings are associated only with paternal, and not maternal, lifespan. Our results therefore support the notion that there are sex differences in the heritability of lifespan. Due to the small study population, we were unable to identify the effects of paternal lifespan on the DNA methylome of daughters and sons separately, although sex was included as a covariate in the regression model. There is a female advantage in longevity, and females have better survival at all ages. Various mechanisms, including hormonal effects and differences in immune function (role of estrogen and androgens, susceptibility to infections [52,53]) as well as the role of X chromosome (skewing of X chromosome inactivation [54]) have been speculated to play a role, but definitive proof is lacking. Similarly, sexual dimorphism in the heritability of factors contributing to lifespan remain to be speculated [55]. Our results also indicate that paternal lifespan is not associated with the socio-economic status of the progeny, suggesting that this observed effect is not due to a shared environment.

## CONCLUSIONS

In summary, we show that length of paternal lifespan is associated with progeny DNA methylation profiles and that this effect can be identified in nonagenarians. To our knowledge, the effects of the full range of parental lifespan on DNA methylation have not been previously analyzed. However, Gentilini et al. [13] did study the effect of extreme longevity in women. The methylation sites associated with paternal lifespan reported in the current study were located in genes associated with development and morphogenesis, as well as cell signaling. These results imply that these processes may be epigenetically regulating lifespan.

These results suggest that part of the “missing” heritability of lifespan may be epigenetic in nature. In addition to epigenetics, rare genetic variants most likely contribute to the heritability of lifespan. Because the length of lifespan is also significantly affected by environmental effects, lifestyle factors and interaction effects between environment and genetics, further studies are needed to uncover the genetic and epigenetic features that provide minor contributions to the heritability of lifespan.

## MATERIALS AND METHODS

### Study population

The study population consisted of 90 individuals born in 1920 (females *n* = 66, males *n* = 24) who participated in the home examinations in the Vitality 90+ Study in the year 2010. The study subjects included in this study were selected from the Vitality 90+ study cohort of 2010 based on two criteria: (i) information on both maternal and paternal lifespan was available and (ii) both parents had a lifespan of 40 years or more. The Vitality 90+ study is an on-going, prospective, population based study that includes both home dwelling and institutionalized individuals who are aged 90 years or more, and who live in the city of Tampere, Finland. The recruitment and characterization of participants were performed as has been reported in earlier Vitality 90+ study cohorts [22]. The study subjects were all of Western European descent and had not had any infections or received any vaccinations in the 30 days prior to blood sample collection. The study participants provided their written informed consent. This study was conducted according to the principles expressed in the declaration of Helsinki, and the study protocol was approved by the ethics committee of the city of Tampere (1592/403/1996).

### Sample collection

Blood samples were collected into EDTA-containing tubes by a trained medical student during a home visit. All blood samples were drawn between 8 am and 12 am. Samples were directly subjected to leucocyte separation on a Ficoll-Paque density gradient (Ficoll-Paque™ Premium, cat. no. 17-5442-03, GE Healthcare Bio-Sciences AB, Uppsala, Sweden). The PBMC layer was collected and was suspended in 1 ml of a freezing solution (5/8 FBS, 2/8 RPMI-160 medium, 1/8 DMSO) (FBS cat. no. F7524, Sigma-Aldrich, MO, USA; RPMI: cat. no. R0883, Sigma-Aldrich, MO, USA; DMSO: cat. no. 1.02931.0500, VWR, Espoo, Finland) and stored in liquid nitrogen.

Information on the age of death of parents and siblings and the age of living siblings was collected with a questionnaire at the home visit.

### DNA extraction

DNA was extracted from PBMCs using the QIAamp DNA Mini kit (Qiagen, CA, USA), following the manufacturer's instructions for the spin protocol. The DNA was eluted in 60 μl of AE elution buffer and stored at −20°C. The concentration and quality of the DNA was assessed with the Qubit dsDNA HS Assay (Invitrogen, Eugene, OR, USA).

### FACS

The proportions of different lymphocyte populations were determined through FACS analysis (BD FACSCanto II), and the results were analyzed with BD FACS Diva, version 6.1.3 (BD Biosciences, Franklin Lakes, NJ, USA). The antibodies employed in this analysis were FITC-CD14 (cat. no. 11-0149), PerCP-Cy5.5-CD3 (45-0037), APC-CD28 (17-0289) (eBioscience, San Diego, CA, USA), PE-Cy™7-CD4 (cat. no. 557852) and APC-Cy™7-CD8 (557834) (BD Biosciences).

### Methylation array

Genome-wide DNA methylation profiling was performed at the Institute for Molecular Medicine Finland (FIMM) Technology Centre of the University of Helsinki in two batches (time interval, 6 months). Bisulfite conversion of 1 μg of DNA was performed using an EZ-96 DNA Methylation Kit (Zymo Research, Irvine, CA, USA) according to the manufacturer's instructions. A 4 μl aliquot of bisulfite-converted DNA was subjected to whole genome amplification and then enzymatically fragmented and hybridized to the Infinium HumanMethylation450 BeadChip (Illumina, San Diego, CA, USA) according to the manufacturer's protocol. Samples were assigned to the arrays in a randomized order. The BeadChips were scanned using an iScan reader (Illumina).

### Processing of the methylation data

The data were processed as described previously [8], and can be accessed in GEO database (GSE58888) [56]. Before any processing, all unspecific or polymorphic sites (*n* = 76775) with minor allele frequency higher than 5%, based on database information [57], and probes mapping to sex chromosomes (*n* = 11 648) were removed. Methylation data were preprocessed as a methylumiset object using R software (R> = 2.15.3) with the wateRmelon array-specific package [58]. Technically poor quality samples and target sites were filtered out by excluding sites with a beadcount of < 3 in 5% of the samples (*n* = 515) and sites for which 1% of the samples had a detection *p*-value > 0.05 (*n* = 698). Background correction and quantile normalization were conducted individually for the two chemistries (Infinium I and II) as well as for the intensities of methylation (m) and un-methylation (u) using the dasen method. After dasen normalization, the u and m intensities were transformed to values of beta (β). β is the ratio of methylated probe (m) intensities to overall intensities (m+u+α), where α is the constant offset, 100. Thus, β ranges linearly between 0 (non-methylated, 0%) and 1 (completely methylated, 100%). Next, the batch effect of the Infinium chemistries was adjusted using the BMIQ algorithm, which is based on beta mixture-models and the EM-algorithm [59]. Several visualization styles were used to verify the quality of the data, including boxplots from the raw intensities, Kernel density plots in the chemistry correction procedure and PCA (principal component analysis).

### Detection of methylation sites associated with parental age

To assess the relationship between site-specific methylation level and the age of the father/mother at the time of death, a generalized regression model, referred to as variable dispersion beta regression [60, 61], was utilized on each CpG site. The age of the father and mother at the time of death (linear variable) and the gender (categories 0 and 1) of the subject were employed as predictors of the site-specific methylation outcome in the form of β-values (ranging from 0 to 1) in each equation, where the mean model with a linker function of logit was utilized. Furthermore, as was previously observed, because methylation levels fluctuate based on the composition of blood cell subtypes [8,62], the variables corresponding to cell type proportions (the CD4+ to CD8+ ratio and the proportions of CD28/CD4+ and CD28-/CD8+ and CD14+ cells) were included as linear covariates in the model. The bias caused by the batch effect of two laboratory days (time interval of 6 months) was also confirmed by PCA. Therefore, a variable corresponding to the batches (categories 0 and 1) was set as covariate in the model. The nominal Benjamini-Hochberg corrected p-value was set to 0.05. Next, the CpG sites with substantial differences in methylation level between the extreme age groups were extracted. The subjects were categorized to groups FI, FII, FIII (and MI, MII, MIII), with equal group sizes according to the age of the father/mother at the time of death (see Table 1). The extraction procedure was conducted by calculating the difference in median values of methylation in each CpG site for groups I and III, and only sites with −0.01>Δβ>0.01 were included for further analysis.

### Pathway analyses

Pathway analyses were performed on genes harboring CpG sites where the level of methylation was associated with paternal lifespan. The 659 CpG sites were located in 422 different genes.

IPA [23] was used to identify canonical pathways associated with the identified genes. According to the manufacturer, these canonical pathways are well characterized metabolic and cell signaling pathways that have been curated and hand-drawn by PhD-level scientists. All of the data sources provided by the Ingenuity Knowledge Base were included in the IPA, and the Ingenuity Knowledge Base was used as the reference set in all analyses. For the association of molecules, only experimentally observed results were accepted, and only human data were considered. A Benjamini-Hochberg multiple testing corrected *p*-value of < 0.05 was used as the threshold for significance. The Ingenuity pathway analysis was performed on the 12^th^ of March 2015.

GOrilla [24, 25] was used to identify the enriched GO terms for the identified genes. GO terms were searched based on two unranked lists (target and background), and all genes with at least one probe in the 450K array were used as the background list. A Benjamini-Hochberg multiple testing corrected *p*-value of < 0.05 was used as the threshold for significance. GOrilla analysis was performed on the 29^th^ of April 2015.

## SUPPLEMENTARY FILES




